# *N*-(2-Hydroxyphenyl)-1-[3-(2-oxo-2,3-dihydro-1*H*- benzimidazol-1-yl)propyl]piperidine-4-Carboxamide (D2AAK4), a Multi-Target Ligand of Aminergic GPCRs, as a Potential Antipsychotic

**DOI:** 10.3390/biom10020349

**Published:** 2020-02-24

**Authors:** Agnieszka A. Kaczor, Katarzyna M. Targowska-Duda, Andrea G. Silva, Magda Kondej, Grażyna Biała, Marián Castro

**Affiliations:** 1 Department of Synthesis and Chemical Technology of Pharmaceutical Substances, Faculty of Pharmacy, Medical University of Lublin, 4A Chodźki St., PL-20093 Lublin, Poland; magda.kondej@onet.pl; 2 School of Pharmacy, University of Eastern Finland, P.O. Box 1627, FI-70211 Kuopio, Finland; 3 Department of Biopharmacy, Faculty of Pharmacy, Medical University of Lublin, 4A Chodźki St., PL-20093 Lublin, Poland; 4 Department of Pharmacology, Universidade de Santiago de Compostela, Center for Research in Molecular Medicine and Chronic Diseases (CIMUS), Avda de Barcelona, E-15782 Santiago de Compostela, Spain; andrea.garcia.silva@rai.usc.es (A.G.S.); marian.castro@usc.es (M.C.); 5 Department of Pharmacology and Pharmacodynamics, Faculty of Pharmacy, Medical University of Lublin, 4A Chodźki St., PL-20093 Lublin, Poland; grazyna.biala@umlub.pl

**Keywords:** antipsychotics, behavioral studies, drug design, in vitro studies, molecular modeling, schizophrenia

## Abstract

*N*-(2-hydroxyphenyl)-1-[3-(2-oxo-2,3-dihydro-1*H*-benzimidazol -1-yl)propyl]piperidine-4-carboxamide (D2AAK4) is a multitarget ligand of aminergic G protein-coupled receptors (GPCRs) identified in structure-based virtual screening. Here we present detailed in vitro, in silico and in vivo investigations of this virtual hit. D2AAK4 has an atypical antipsychotic profile and low affinity to off-targets. It interacts with aminergic GPCRs, forming an electrostatic interaction between its protonatable nitrogen atom and the conserved Asp 3.32 of the receptors. At the dose of 100 mg/kg D2AAK4 decreases amphetamine-induced hyperactivity predictive of antipsychotic activity, improves memory consolidation in passive avoidance test and has anxiogenic properties in elevated plus maze test (EPM). Further optimization of the virtual hit D2AAK4 will be aimed to balance its multitarget profile and to obtain analogs with anxiolytic activity.

## 1. Introduction

Schizophrenia is a severe and chronic mental illness with high prevalence (appearing in about 0.5%–1% of the population) [1], and it remains among the 20 leading causes of health loss in terms of years lived with disability (YLDs) from 161 causes analyzed worldwide [2]. Symptoms of schizophrenia generally emerge during adolescence or early adulthood and are grouped into positive, negative and cognitive symptoms. Psychotic or positive symptoms include severe thought disorganization like hallucinations (often auditory hallucinations) and delusions (which often involve persecution or megalomania). Deficit or negative symptoms include affect flattening, poverty of speech, social withdrawal and anhedonia. Cognitive symptoms concern memory and concentration disturbances.

The pathomechanism of schizophrenia remains unclear, but it is generally agreed that it involves many neurotransmitter systems. The central hypothesis of schizophrenia is still the dopaminergic hypothesis, which is complemented by the glutamatergic hypothesis [3,4,5]. Other neurotransmitter systems, in particular serotonin and γ-aminobutyric acid (GABA), as well as cholinergic, cannabinoid and opioid systems, are also important in the pathogenesis of schizophrenia.

Antipsychotic drugs are crucial for the comprehensive approach to the treatment of schizophrenia as they are able to supply significant symptom relief and a broad psychosocial recovery for patients. Although modern antipsychotics do not cure schizophrenia, these drugs control the intensity of the symptoms, diminish exacerbations of the illness and decrease the risk of relapse [6]. Nevertheless, current antipsychotic drug therapies are associated with significant side effects and medical morbidity burdens that may hinder progress toward treatment goals.

The drugs used in schizophrenia are divided into typical (first generation) and atypical antipsychotics, and among the latter, there are drugs classified as second and third generation. This classification groups together drugs rather heterogeneous in their clinical efficacy and tolerability as well as pharmacological profile [7]. However, it takes into account some fundamental mechanisms of action that are considered essential for the antipsychotic efficacy of currently approved antipsychotics. Both first- and second-generation antipsychotics present a dopamine D_2_ antagonist component in their pharmacological profile, which, in the case of second-generation atypical antipsychotics, can be of weak affinity (e.g., clozapine, quetiapine) and it is complemented with a serotonin 5-HT_2A_ antagonism of relative higher affinity. Chronic blockade of striatal D_2_ receptors and high occupancy of the receptor in the striatum have been related to extrapyramidal symptoms that constitute the more severe adverse effect of typical antipsychotics, whereas the combined D_2_/5-HT_2A_ antagonism that characterizes second-generation drugs would help to avoid those serious side effects of typical neuroleptics, i.e., motor disorders from the extrapyramidal system. Finally, drugs such as aripiprazole, brexpiprazole and cariprazine, are classified as third-generation antipsychotics, and they are considered partial or biased agonists of dopamine D_2_ receptor. Still, atypical antipsychotics modulate a broader panel of targets that exceed those mentioned above, constituting a prototypical example of multitarget therapies, and their complex modulation of neurotransmitter receptors beyond D_2_ and 5-HT_2A_, together with additional actions on neuronal plasticity and neurogenesis, in the long term might contribute to clinical efficacy [7]. In spite of the broad pharmacotherapeutic arsenal currently approved, there is still no antipsychotic medication efficacious against all the different symptoms of schizophrenia, and an improved safety profile of these drugs is still highly desirable.

The change in the paradigm of “one disease, one gene, one goal, one drug” prevailing over the last 20 years in the chemistry of drugs is shifting the therapeutic process towards drugs affecting many molecular targets, i.e., multitarget drugs that have become a hope in the treatment of diseases with complex pathomechanism such as schizophrenia. Multitarget drugs work comprehensively because they take into account the complexity of the molecular mechanism of the disease and give a better chance to modify or cure the disease process. In addition, a single multitarget drug offers less risk of drug–drug interactions, which is a significant advantage over combination therapies. Administration of one single drug is more convenient, reducing the risk of incorrect dosage or medication noncompliance of more complex dosage regimens. Another benefit would be the eventually fewer side effects [8].

**Figure 1:**
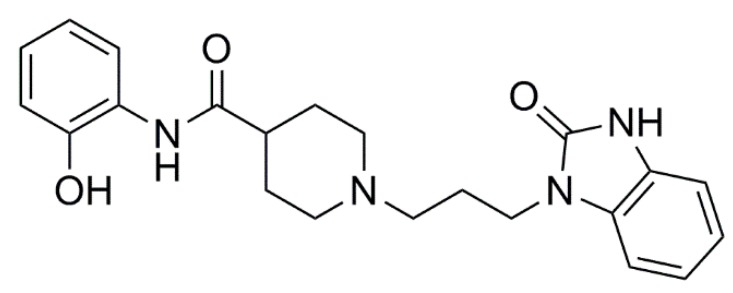
The virtual hit *N*-(2-hydroxyphenyl)-1-[3-(2-oxo-2,3-dihydro-1*H*-benzimidazol-1-yl)propyl]piperidine-4-carboxamide (D2AAK4).

The aim of this work was to use molecular modeling, in vitro assays and behavioral studies to evaluate the new compound *N*-(2-hydroxyphenyl)-1-[3-(2-oxo-2,3-dihydro-1*H*-benzimidazol -1-yl)propyl]piperidine-4-carboxamide (D2AAK4), Figure 1 which was identified in structure-based virtual screening aimed to search for novel ligands of dopamine D_2_ receptor [9]. The virtual screening was performed using dopamine D_2_ receptor complexes with chlorprothixene and olanzapine obtained by induced fit docking approach. As the olanzapine is an atypical antipsychotic, the binding pocket was modified to prioritize compounds with the atypical profile. It should be stressed, however, that in order to identify atypical antipsychotics with higher affinity to serotonin 5-HT_1A_ and 5-HT_2A_ receptors than to dopamine D_2_ receptors, serotonin receptors should be also considered during the virtual screening campaign, not to limit the study. This will be the aim of our future work. Nevertheless, D2AAK4, identified in the previous virtual screening experiment, is a multitarget ligand with affinity to D_2_ receptor, additional affinity for D_1_, D_3_, 5-HT_2A_ and 5-HT_7_ receptors and with low affinity for potential off-targets M_1_ and H_1_ receptors. We used here a combination of in vitro, in silico (homology modeling, molecular docking, molecular dynamics) and in vivo approaches (to assess antipsychotic, procognitive and anxiolytic properties of the compound) to characterize the virtual hit D2AAK4 before its future rational optimization as lead structure for a potential antipsychotic.

## 2. Materials and Methods

### 2.1. In Vitro Studies

#### 2.1.1. Receptor Binding Assays

Radioligand binding assays were performed on cell membrane preparations from cell lines stably expressing the human cloned receptors. Chinese hamster ovary K1 (CHO-K1) cell lines stably expressing human D_2S_, 5-HT_2A_ or H_1_ receptors and human embryonic kidney 293 (HEK293) cell lines stably expressing human 5-HT_1A_ or 5-HT_7(a)_ receptors were in-house available [9,10,11], whereas CHO-K1 cell lines stably expressing human D_1_ or D_3_ receptors (Perkin Elmer, Waltham, MA, USA) and Chem-1 cell line stably expressing human M_1_ receptor (Millipore, Merck KGaA, Darmstadt, Germany) were commercially available. Experimental conditions for binding assays at D_1_, D_2_, D_3_, 5-HT_1A_ and 5-HT_2A_ receptors have been previously reported [12]. 0.7 nM [^3^H]-SCH23390, 2 nM [^3^H]-SB269970 (5-HT_7_), 2 nM [^3^H]-Pyrilamine (H_1_) and 2 nM [^3^H]-Pirenzepine (M_1_) were employed as radioligands. Nonspecific binding was assessed in the presence of 25 µM clozapine (5-HT_7_), 10 µM triprolidine (H_1_) and 200 µM pirenzepine (M_1_). D2AAK4 was initially evaluated at the single concentration (10 µM) in competition radioligand binding assays at all the different receptors studied, and at those receptors at which 10 µM compound achieved >40% inhibition of specific radioligand binding, competition binding curves of D2AAK4 were constructed using six or seven different concentrations of compound, starting at 0.1 nM or 1 nM till full displacement or a maximal concentration (100 µM or 300 µM) was reached. Affinity (equilibrium dissociation constant (*K*_i_) and p*K*_i_ (−log*K*_i_)) values were calculated as previously reported [13]. Affinity values of reference compounds that were assayed in parallel to D2AAK4 as internal controls in the binding assays are given in Supplementary Figure S1 (legend).

#### 2.1.2. Functional Assay at D_2_ Receptors

The efficacy of D2AAK4 as agonist or antagonist of D_2_ receptors was evaluated in functional assays of cAMP signaling in the CHO-K1 cell line stably expressing the human D_2S_ receptor employed in radioligand binding assays, following a protocol already described [9,12]. Compound was initially assessed at 10 µM concentration either as agonist or as antagonist of 10 µM dopamine response. Dopamine (10 nM–1 mM) was used as reference agonist in these assays. The antagonist effect of D2AAK4 on 10 µM dopamine response was further investigated in concentration-response curves constructed using seven concentrations of D2AAK4 ranging from 0.1 nM to 100 µM. Haloperidol (0.1 nM–10 µM) was used as reference antagonist in these assays.

#### 2.1.3. Functional Assay at 5-HT_2A_ Receptors

The efficacy of D2AAK4 as agonist or antagonist of 5-HT_2A_ receptors was evaluated in functional assays of inositol phosphate (IP) production in the CHO-K1 cell line stably expressing the human 5-HT_2A_ receptor employed in radioligand binding assays, following a protocol already described [11]. Compound was assessed in concentration (0.1 nM–100 µM)-response curves either as agonist or as antagonist of 1 µM serotonin (5-HT) response. 5-HT (0.1 nM–100 µM) and risperidone (0.01 nM–10 µM) were used in these assays as reference agonist and antagonist, respectively.

### 2.2. Molecular Modeling

#### 2.2.1. Receptor Structures

In cases where receptor X-ray structures were available, they were taken for molecular docking after necessary mutations and preparation with Schrödinger software v. 2019-4 [14]: dopamine D_2_ receptor in complex with the antagonist risperidone (PDB ID: 6CM4 [15]), dopamine D_3_ receptor in complex with the antagonist eticlopride (PDB ID: 3PBL [16]) and serotonin 5-HT_2A_ receptor in complex with the antagonist risperidone (PDB ID: 6A93 [17]. For dopamine D_1_ receptor, a previously reported homology model [12] was used. For serotonin 5-HT_7_ receptor, a homology model was constructed with Modeller v. 9.19 [18] using turkey β_1_ adrenoreceptor in complex with the antagonist cyanopindolol (PDB ID: 2YCX [19]) as a template. All receptor structures were in inactive conformation, as D2AAK4 is or is expected to be their antagonist. MUSCLE (MUltiple Sequence Comparison by Log- Expectation) was used for sequence alignment [20]. The sequence identity was 39% and sequence similarity was 58%. A population of 100 models was generated. The best model was selected based on Discrete Optimized Protein Energy (DOPE) profiles obtained from Modeller v. 9.19 software.

#### 2.2.2. Compound Preparation

D2AAK4 was modeled using LigPrep module [21] of Schrödinger suite of software, v. 2019-4 as previously reported [9,12,13,22,23]. In order to identify the protonation state, Epik module [24] of Schrödinger suite of software, v. 2019-4 was applied.

#### 2.2.3. Molecular Docking

Standard Precision (SP) approach of Glide [25] from Schrödinger release 2019-4 was used for molecular docking of D2AAK4 to receptor models as reported previously [9,12,13,22,23]. 100 poses were generated for each receptor. The final poses were selected based on Glide docking scores, and visual inspection among the poses where the protonatable nitrogen atom of the ligand interacted with conserved Asp 3.32 (number indicates Ballesteros-Weinstein nomenclature [26]). Visualization of molecular modeling results was achieved with Maestro Release 2019.4 [14] and PyMol 2.0.4 [27] software.

#### 2.2.4. Molecular Dynamics

The D2AAK4-receptor complexes were subjected to molecular dynamics with Desmond v. 3.0.3.1 [28] as reported previously [12,22]. In short, the complexes were immersed in POPC (1-palmitoyl-2-oleoyl-sn-glycero-3-phosphocholine) membrane and hydrated, and ions were added to neutralize protein charges and then to the concentration of 0.15 M NaCl. The complexes were minimized and subjected to MD first in the NVT ensemble for 1 ns and then in NPT ensemble for 20 ns with the restrictions on the protein backbone in each case. The production runs were performed in NPT ensemble with no restrictions for 200 ns.

### 2.3. Behavioral Studies

#### 2.3.1. Animals

The experiments were carried out on naive Swiss male mice (Farm of Laboratory Animals, Warszawa, Poland), 2 months old and weighing 20–30 g. All experiments were performed according to the National Institute of Health Guidelines for the Care and Use of Laboratory Animals and the European Community Council Directive for Care and Use of Laboratory Animals (2010/63/EU) and approved by the local ethics committee (license 2/2015) as previously described [12]. Mice were maintained under standard laboratory conditions and were adapted to the laboratory conditions as described in our earlier work [12].

#### 2.3.2. Drugs

D2AAK4 was purchased from Enamine (Z126202476), d-amphetamine sulphate and saline (0.9% NaCl) from Sigma-Aldrich (St. Louis, MO, USA). D2AAK4 was suspended in 1% Tween 80 and then diluted in saline (0.9%, vehicle) and d-amphetamine was dissolved in saline. The agents were administered intraperitoneally (i.p.) as a 10 mL/kg volume. D2AAK4, d-amphetamine and vehicle were freshly prepared before each experiment. Control groups received injections (vehicle) at the same volume as well as by the same route of administration.

#### 2.3.3. Motor Coordination Evaluated by Rotarod And Chimney Tests

Motor coordination has been assessed in mice using the rotarod test and the chimney test using the same procedure as described in our earlier work [12]. Both tests were performed 60 min after injection of D2AAK4 (100 mg/kg; i.p.) (n = 8) or vehicle (i.p.) (n = 8), using the same mice (i.e., after rotarod test each mouse was placed to the chimney test). D2AAK1 was previously studied using the same dose so we decided to select the dose of 100 mg/kg.

#### 2.3.4. Spontaneous Locomotor Activity and Amphetamine-Induced Hyperactivity

The effect of D2AAK4 on mouse spontaneous locomotor activity was measured using an animal activity meter Opto-Varimex-4 Auto-Track (Columbus Instruments, USA) kept in a sound-attenuated experimental room. The Auto-Track System senses motion with a grid of infrared photocells monitoring movements of animals placed in the transparent cages. More specifically, the arithmetic average for distance (cm) traveled by a mouse (± SEM) measured for each group was analyzed and compared to respective controls. Each mouse was placed for 30 min test to each cage 60 min after D2AAK4 (100 mg/kg; i.p.) (n = 7), amphetamine (5 mg/kg; s.c.) (n = 8) and vehicle (as a control) (n = 8) administration. To determine whether D2AAK4 influences amphetamine-induced hyperactivity, each animal was injected with D2AAK4 (100 mg/kg; i.p.) or vehicle (n = 7–8) and 30 min later mice were treated with amphetamine (5 mg/kg; s.c.) or saline (n = 7–8). Then, 30 min later, spontaneous locomotor activity was measured.

#### 2.3.5. Passive Avoidance Task

The effect of D2AAK4 on long-term memory was evaluated in mice using the passive avoidance (PA) task. The PA apparatus was described previously [12]. The PA procedure was also previously described [12,29].

The experimental procedure involved evaluation of the memory consolidation after D2AAK4 (100 mg/kg; i.p.) or vehicle (control group) injections. Mice received D2AAK4 immediately after being pretested on Day 1, and then they were retested (without injections) 24 h later. The changes in PA performance were calculated as the difference between retention and training latencies (i.e., latency index (IL)). More specifically, IL was calculated for each animal and presented as the ratio (as described in [12,29]).

#### 2.3.6. Elevated Plus Maze (EPM) Procedure

The effect of D2AAK4 on mouse anxiety-like behavior was evaluated using the elevated plus maze (EPM). The EPM apparatus was used as described previously [12]. The EPM procedure was described in our previous papers [12,30]. The percentage (%) of time spent in the open arms and % of entries into the open arms were calculated. Moreover, the number of entries into the closed arms was recorded as the motor activity indicator of tested mice.

To determine D2AAK4 acute effect on anxiety-like behavior, the studied compound (100 mg/kg; i.p.) (n = 8) or vehicle (i.p.) (n = 8) was injected 30 and 60 min before the EPM test.

#### 2.3.7. Statistical Analysis

The statistical analyses were performed by two-way analysis of variance (ANOVA), followed by the Tukey’s post hoc test to compare the pretreatment and treatment effects. One-way ANOVA and t-test were used to compare differences between drugs and control group. Data were presented as mean ± SEM. The confidence limit of p<0.05 was considered statistically significant.

## 3. Results

### 3.1. In Vitro Studies

#### 3.1.1. Affinity Profile of D2AAK4 at Selected Targets

In a previous study, we carried out a structure-based virtual screening that resulted in the identification of the compound D2AAK4, among other novel dopamine D_2_ receptor ligands. The pharmacological profiling of D2AAK4 on a selected panel of dopamine D_1_-like (D_1_) and D_2_-like (D_2_, D_3_), as well as serotonin (5-HT_1A_ and 5-HT_2A_), receptors led to its characterization as a low-affinity D_2_ receptor antagonist with moderate (3.67 times higher) affinity for 5-HT_2A_ receptors [9] (Table 1), being a 5-HT_2A_/D_2_ affinity ratio (*K*_i_ D_2_/*K*_i_ 5-HT_2A_) higher or equal to 1.12 (Meltzer’s ratio), considered relevant for antipsychotic clinical efficacy and safety [31]. Here, we aimed at extending the in vitro pharmacological affinity profiling of D2AAK4 to other receptors from the GPCR receptorome considered relevant for clinical efficacy and/or side effects of antipsychotics, that is, serotonin 5-HT_7_, histamine H_1_ and muscarinic M_1_ receptors. The results from radioligand binding assays revealed the low affinity of D2AAK4 for these three targets. Affinity values (p*K*_i_, *K*_i_) of D2AAK4 at the selected panel of targets (except for 5-HT_1A_, at which D2AAK4 did not show affinity (% inh. at 10 μM (mean ± SEM) = 9.7 ± 0.4%)) are given in Table 1. Examples of competition radioligand binding curves of D2AAK4 at the different receptors profiled can be found in Supplementary Information (Figure S1).

#### 3.1.2. D2AAK4 Is a D_2_/5-HT_2A_ Antagonist

**Figure 2:**
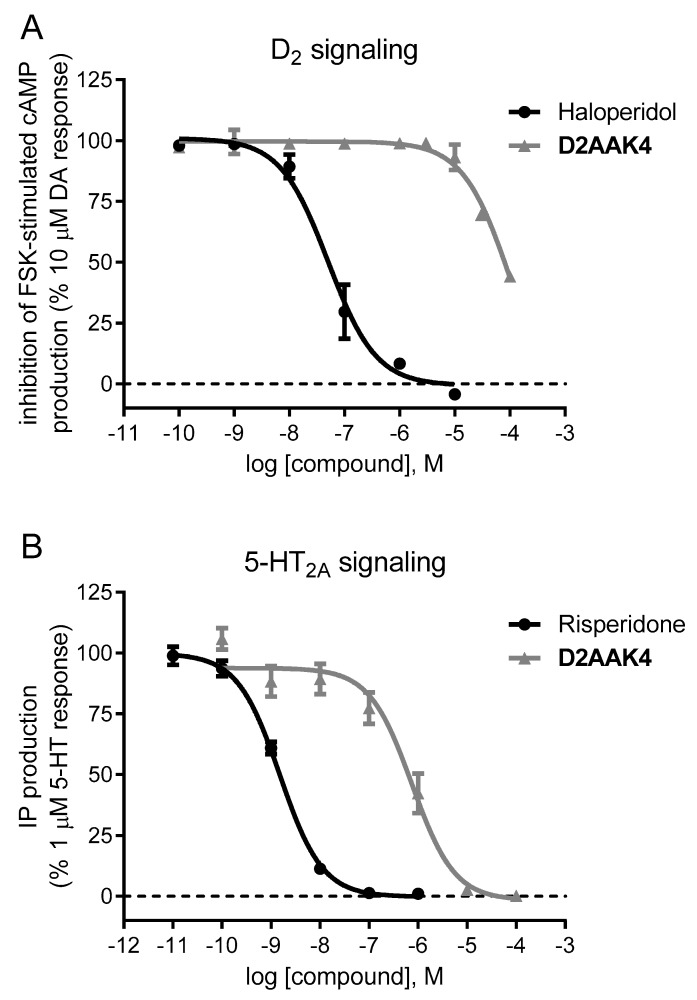
Functional assays for D2AAK4 at D_2_ and 5-HT_2A_ receptors. **A**—Concentration-response curves of D2AAK4 and haloperidol (as reference antagonist) on the inhibition of forskolin (FSK)-stimulated cAMP production by 10 µM dopamine (DA) in CHO-K1 cells expressing human-cloned D_2_ receptors. The graph shows data (mean ± SEM) of two independent experiments performed in duplicate. **B**—Concentration-response curves of D2AAK4 and risperidone (as reference antagonist) on IP production stimulated by 1 µM 5-HT in CHO-K1 cells expressing human cloned 5-HT_2A_ receptors. The graph shows data (mean ± SEM) of three independent experiments performed in duplicate or triplicate.

In a previous work, we characterized D2AAK4 as a D_2_ antagonist at the cAMP pathway in vitro second messenger assays (inhibition of forskolin-stimulated cAMP production) carried out in CHO-K1 cells stably transfected with human D_2_ receptor, where 10 µM D2AAK4 antagonized by 12% the response of 10 µM dopamine [9] (Supplementary Information, Table S1). The antagonist efficacy of D2AAK4 at D_2_ receptors was confirmed in assays of cAMP production by constructing concentration-response curves of the compound (Figure 2A). D2AAK4 antagonized dopamine response in a concentration-dependent manner to an extent of 44% at 100 µM concentration, the maximal assayed (Figure 2A and Supplementary Information, Table S1). IC_50_ of reference D_2_ antagonist haloperidol was 56 nM in these assays (pIC_50_ (mean ± SEM) = 7.25 ± 0.21) (Figure 2A).

5-HT_2A_ antagonism combined with weaker D_2_ antagonism is considered a key feature in the pharmacological profile of atypical antipsychotics [33]. Therefore, the efficacy of D2AAK4 at human 5-HT_2A_ receptors was further evaluated in functionally in vitro assays of second messenger (inositol phosphate) production in CHO-K1 cells stably expressing the receptor. The compound was unable to promote IP signaling in the range of concentrations assayed (from 0.1 nM to 100 µM), indicating lack of agonist efficacy, while the control agonist 5-HT efficiently stimulated IP signaling in dose-response curves under the same conditions (data not shown). On the contrary, 1 μM 5-HT-stimulated IP production was fully antagonized by D2AAK4 in concentration (0.1 nM–100 µM)-response curves, showing an IC_50_ value of 714 nM consistent with its affinity value (Figure 2B and Supplementary Information, Table S1). IC_50_ of reference 5-HT_2A_ antagonist risperidone was 1.5 nM in these assays (pIC_50_ (mean ± SEM) = 8.82 ± 0.05) (Figure 2B).

### 3.2. Molecular Modeling

#### 3.2.1. Receptor Structures

**Figure 3:**
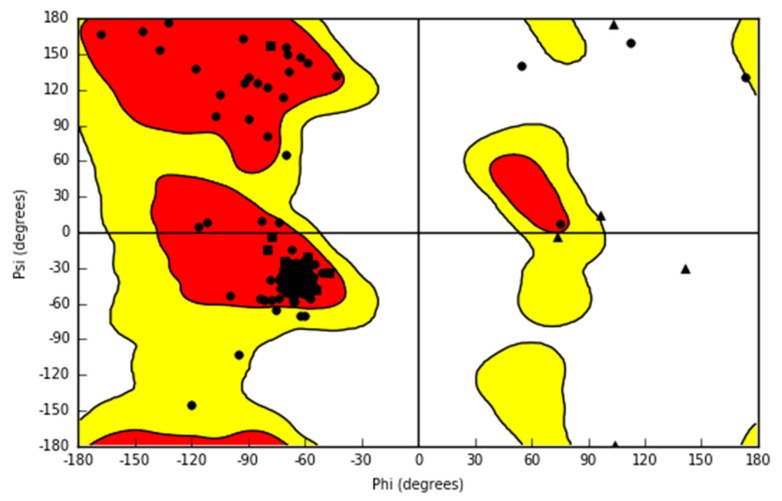
The Ramachandran plot of the final homology model of serotonin 5-HT_7_ receptor. Most residues are represented by circles. Glycines are shown as triangles. Prolines are shown as squares. Two outliers are Asn 226 and Ile 353, which are in extracellular loop regions.

As stated in Materials and Methods section, in the current study, we used previously published model of D_1_ receptor [12] and X-ray structures of D_2_ [15], D_3_ [16] and 5-HT_2A_ [17] receptors. Here, we report a new homology model of 5-HT_7_ receptor. The Ramachandran plot of the final homology model of serotonin 5-HT_7_ receptor is shown in Figure 3. The plot confirms the good quality of the constructed homology model. The majority of residues lie in the allowed regions. The only outliers, not counting proline and glycine residues, are Asn 226 and Ile 353 from extracellular loop regions. The model was compared with the 5-HT_7_ receptor homology model in inactive conformation downloaded from GPCRdb [34]. The RMSD (root-mean-square deviation) measured for complete models (including loops) was 1.7 Å.

#### 3.2.2. Molecular Interactions of D2AAK4 with Aminergic G Protein-Coupled Receptors (GPCRs)

**Figure 4:**
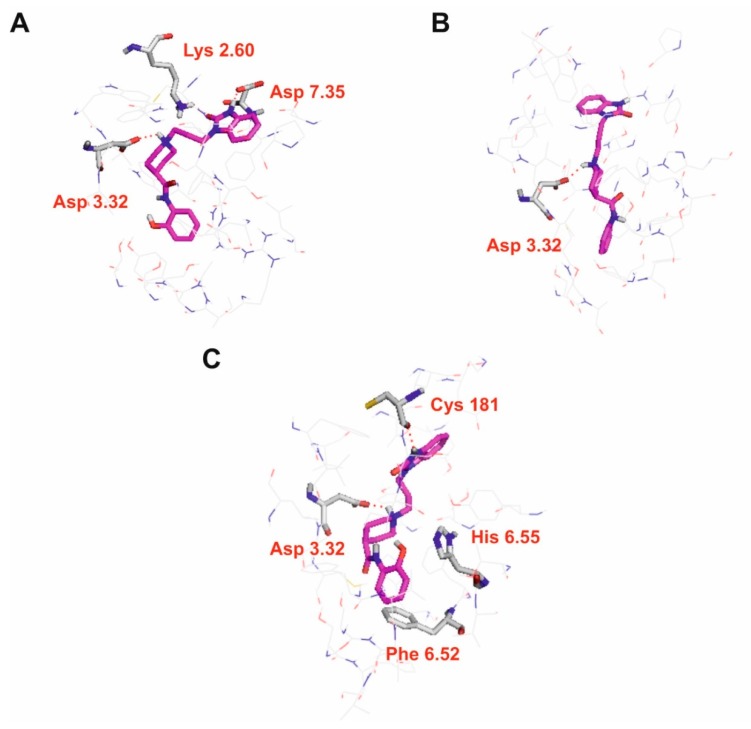
Molecular interactions of D2AAK4 with dopamine D_1_ (**A**), D_2_ (**B**) and D_3_ (**C**) receptors. Ligands are represented as sticks with magenta carbon atoms. Protein represented as wire with grey carbon atoms, and main interacting residues are shown as sticks. Hydrogen bonds are shown as red dashed lines. Nonpolar hydrogen atoms not shown for clarity.

**Figure 5:**
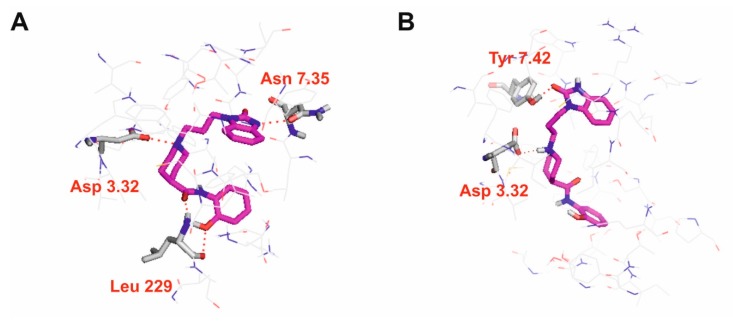
Molecular interactions of D2AAK4 with serotonin 5-HT_2A_ (**A**) and 5-HT_7_ (**B**) receptors. Ligands are represented as sticks with magenta carbon atoms. Protein represented as wire with grey carbon atoms, main interacting residues, are shown as sticks. Hydrogen bonds are shown as red dashed lines. Nonpolar hydrogen atoms are not shown for clarity.

In order to study molecular interactions of D2AAK4 with dopaminergic and serotoninergic receptors, molecular docking was performed. Figure 4 presents interactions of D2AAK4 with dopamine D_1_, D_2_ and D_3_ receptors, while Figure 5 presents interactions with serotonin 5-HT_2A_ and 5-HT_7_ receptors. The binding pose of D2AAK4 with all aminergic GPCRs studied is similar in all receptors. The main anchoring point is the conserved Asp 3.32 [26] from the third transmembrane helix, which forms an electrostatic interaction with the protonatable nitrogen atom of the ligand. 2-Oxo-2,3-dihydro-1*H*-benzimidazolyl moiety of D2AAK4 directs towards the receptor extracellular vestibule while 2-hydroxyphenyl group is located deeper in the receptor cavity. In the predicted binding pose of D2AAK4 in the D_2_ receptor (Figure 4B), the amide group of D2AAK4 is in the (strained) cis conformation. This conformation was found using flexible docking with Glide. It should be stressed that in all the poses of D2AAK4 in the D_2_ receptor which involved the salt bridge with Asp 3.32 contained no steric clashes and were in general in accordance with the poses of this compound with other receptors studied, the amide group was in cis configurations. The highest scored pose was selected from them. In the case of interactions of D2AAK4 with dopamine D_1_ receptor (Figure 4A), the ligand forms hydrogen bonds between its 2-oxo-2,3-dihydro-1*H*-benzimidazolyl group and side chains of Lys 2.60 and Asp 7.35. In the case of dopamine D_3_ receptor, the 2-oxo-2,3-dihydro-1*H*-benzimidazolyl group of the ligand forms a hydrogen bond with the main chain of Cys 181 from the second extracellular loop (Figure 4C). Moreover, π–π stacking interactions between the 2-hydroxyphenyl group of the ligand and Phe 6.52 and His 6.55 were found for this receptor.

In case of interactions of D2AAK4 with serotonin 5-HT_2A_ receptor 2-oxo-2,3-dihydro-1*H*-benzimidazolyl group of the ligand forms a hydrogen bond with the side chain of Asn 7.35 (Figure 5A). Furthermore, hydroxyl group of the ligand, as well as its amide oxygen atom, form hydrogen bonds with the main chain of Leu 229 from the second extracellular loop. For interactions of D2AAK4 with serotonin 5-HT_7_ receptor, a hydrogen bond between the 2-oxo-2,3-dihydro-1*H*-benzimidazolyl group of the ligand and a side chain of Tyr 7.42 was found.

#### 3.2.3. Dynamic Aspects of Interactions of D2AAK4 with Aminergic GPCRs

200 ns molecular dynamics simulations were performed to study dynamic aspects of ligand-receptor interactions. Ligand RMSD values plots are presented in Figure S2 in Supplementary Information proving the correctness of the performed simulations and the stability of the systems.

**Figure 6:**
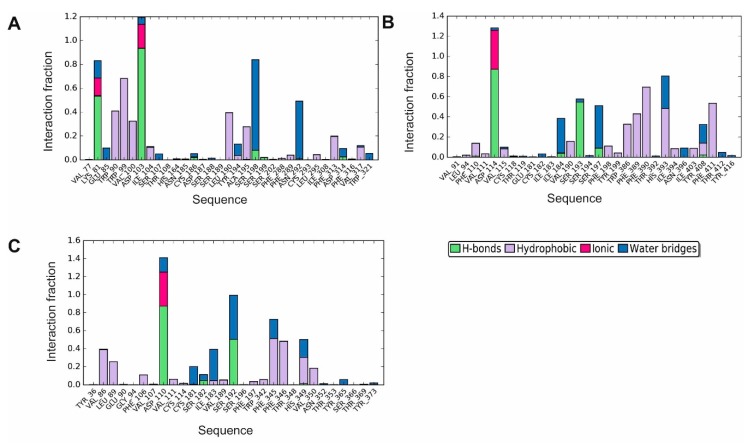
Molecular interactions of D2AAK4 with human dopamine D_1_ (**A**), D_2_ (**B**) and D_3_ (**C**) receptor during 200 ns molecular dynamics simulations are shown as histograms of interactions. The stacked bar charts are normalized over the course of the trajectory: for example, a value of 0.7 suggests that for 70% of the simulation time the specific interaction is maintained. Values over 1.0 are possible as some protein residue may make multiple contacts of the same subtype with the ligand.

**Figure 7:**
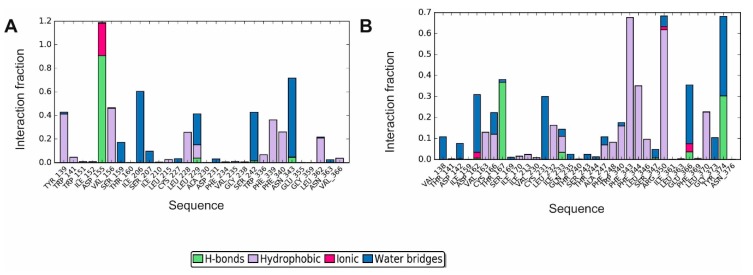
Molecular interactions of D2AAK4 with human serotonin 5-HT_2A_ (**A**) and 5-HT_7_ (**B**) receptor during 200 ns molecular dynamics simulations: histogram of interactions. The stacked bar charts are normalized over the course of the trajectory: for example, a value of 0.7 suggests that for 70% of the simulation time the specific interaction is maintained. Values over 1.0 are possible as some protein residue may make multiple contacts of the same subtype with the ligand.

In order to study D2AAK4 interactions with aminergic GPCRs during 200 ns molecular dynamics simulations, histograms and summary plots of interactions were generated (Figure 6, Figure 7, Figures S3 and S4). In case of all dopamine receptors studied, the contact (ionic or hydrogen bond) with the conserved Asp 3.32 is maintained during 100% of simulations time. Hydrogen bond (or electrostatic or water bridges) interactions between D2AAK4 and Lys 81 (2.60) of dopamine D_1_ receptor were found during 81% of simulations. Water-mediated polar contacts were also identified between the ligand and Ser 198 (5.43) and Asn 292 (6.55) of the receptor (67% and 39% of simulations time, respectively). D2AAK4 was also involved with hydrophobic interactions with Trp 90 (first extracellular loop), Trp 99 (3.28), Val 100 (3.29) and Leu 190 (second extracellular loop).

Regarding interactions of D2AAK4 with dopamine D_2_ receptor, the ligand mediates hydrogen bond interactions with Ser 193 (5.43) during 54% of simulations time and π–π stacking interactions with Phe 390 (6.52) during 38% of simulations time. Hydrophobic interactions were also found between the ligand and Trp 386 (6.48), Phe 389 (6.51), Phe 390 (6.52), His 393 (6.55) and Phe 411 (7.37).

In case of interactions of D2AAK4 with dopamine D_3_ receptor hydrogen bond between the ligand and Ser 192 (5.43) was maintained during 48% of simulations time. Additional residues were involved in hydrophobic interactions: Val 86 (2.60), Phe 345 (6.51), Phe 346 (6.52) and His 349 (6.55).

Studying dynamic aspects of interactions of D2AAK4 with serotonin 5-HT_2A_ and 5-HT_7_ receptors reveals that the electrostatic or hydrogen bond interaction with the conserved Asp 3.32 is maintained for 100% of simulations time for 5-HT_2A_ receptor, while it is mainly a water bridge existing through about 30% of simulations time for 5-HT_7_ receptor. In the case of 5-HT_2A_ receptor, additional water-mediated polar contacts between the ligand and Ile 206 (4.56) or Ser 242 (5.46) [34] were formed during 58% and 30% of simulations time, respectively. In the case of 5-HT_2A_ receptor, the following residues were involved in hydrophobic interactions: Trp 139 (first extracellular loop), Val 156 (3.33), Leu 228 (second extracellular loop), Phe 339 (6.51), Phe 340 (6.52) and Leu 362 (7.34).

In the case of 5-HT_7_ receptor, D2AAK4 forms a hydrogen bond with Thr 167 (3.37) and Tyr 374 (7.42) during 36% and 30% of simulations time, respectively. Moreover, the ligand mediates cation π interactions with Arg 350 (6.58). The residues involved in hydrophobic interactions are Phe 343 (6.51), Phe 344 (6.52) and Arg 350 (6.58).

### 3.3. Behavioral Studies

#### 3.3.1. Effects of D2AAK4 on Motor Coordination, Spontaneous Locomotor Activity and Amphetamine-Induced Hyperactivity in Mice

**Figure 8:**
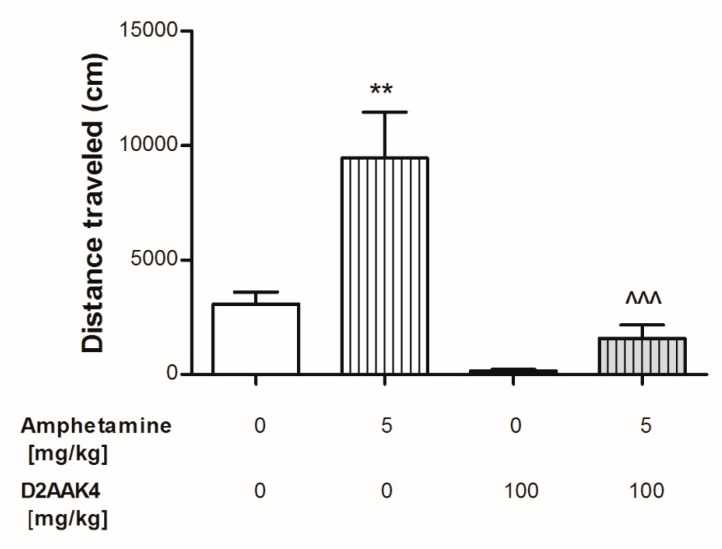
Acute effect of D2AAK4 on the amphetamine-induced hyperactivity in mice. Appropriate groups of mice received D2AAK4 ((100 mg/kg; i.p. (n = 7)), amphetamine (5 mg/kg, (n = 8); s.c.), D2AAK4 (100 mg/kg) coadministered with 5 mg/kg amphetamine (n = 7) and vehicle (n = 8, indicated as 0). Data are presented as mean ± SEM of the distance traveled (cm) by mouse recorded for 30 min. The post hoc Tukey’s test indicates: ** *p* < 0.01 amphetamine vs. the vehicle-treated group and ^^^ *p* < 0.001 D2AAK4 coadministered with amphetamine vs. amphetamine-treated group.

D2AAK4 at the studied dose (100 mg/kg, i.p.) does not impair mouse motor coordination as evaluated in the rotarod and chimney tests (data not shown). The dose was selected based on the previous results for D2AAK1 compound [12]. Lower doses have not been tested to reduce the number of animals used, and this is a limitation to this actual study. However, in the future, we are planning to repeat the same behavioral studies at different doses. The influence of D2AAK4 on the spontaneous locomotor activity in mice is presented in Figure 8. One-way ANOVA analyses revealed significant changes in the locomotor activity after D2AAK4 (100 mg/kg) and amphetamine (5 mg/kg) treatment in mice (F(2,18) = 15.72, *p* = 0.0001). In addition, the post hoc Tukey’s test showed a statistically significant increase in mice locomotor activity after amphetamine treatment (*p* < 0.01). We also determined the effect of D2AAK4 on amphetamine-induced hyperactivity. Two-way ANOVA revealed significant pretreatment (D2AAK4 or vehicle) x treatment (amphetamine or vehicle) interaction (F(1,26) = 6.14, *p* = 0.0200), significant pretreatment effect (F(1,26) = 29.04, *p* < 0.0001) as well as treatment effect (F(1,26) = 15.07, *p* = 0.0006). In addition, the post hoc Tukey’s test showed that administration of amphetamine (*p* < 0.01, Figure 8) increased the locomotor activity of mice, while D2AAK4 did not significantly change locomotor activity in mice when compared to control group. However, D2AAK4 coadministered with amphetamine decreased amphetamine-induced hyperactivity when compared to the amphetamine-treated group (*p* < 0.001, Figure 8).

#### 3.3.2. Effect of Acute Administration of D2AAK4 on Memory Consolidation in Mice

**Figure 9:**
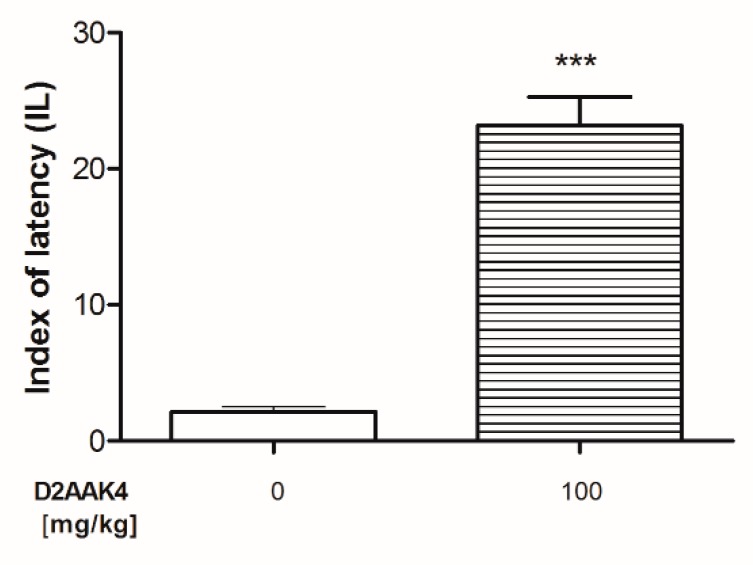
Acute effect of D2AAK4 on memory consolidation in mice evaluated in passive avoidance (PA) test. Appropriate groups of mice were injected D2AAK4 (100 mg/kg (n = 8); i.p.) and vehicle ((n = 8, indicated as 0); i.p.) on Day 1 immediately after the PA test, and 24 h later (i.e., on Day 2), these mice were retested as previously described in [12]. Data are presented as mean ± SEM. The results from the t-test analyses indicate: *** *p* < 0.0001 when compared with the vehicle-treated group.

The D2AAK4 effect on memory consolidation was evaluated during the retention trial of the PA task (Figure 9). T-test revealed that the acute administration of D2AAK4 (100 mg/kg; i.p.) induces a statistically significant increase in the IL values (*p* < 0.0001) compared to respective-vehicle treated group. More specifically, the enhancement of the memory consolidation was observed after D2AAK4 treatment.

#### 3.3.3. Anxiety-Like Effects after D2AAK4 Treatment in Mice

**Figure 10:**
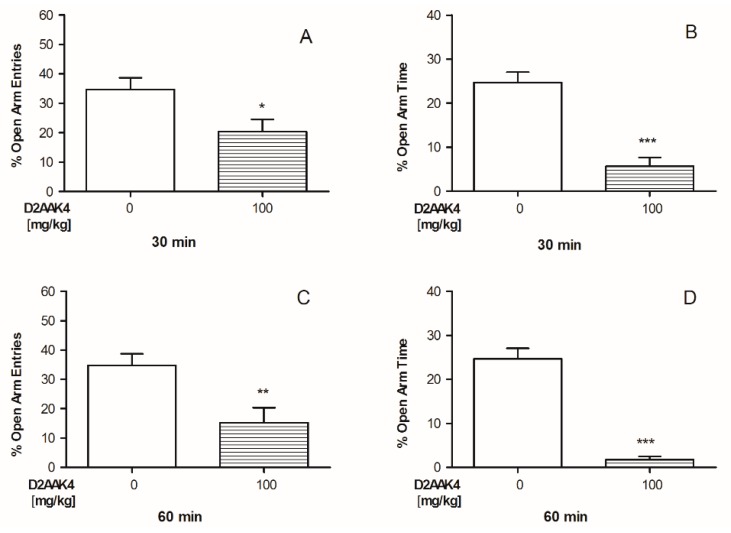
Acute effect of D2AAK4 on anxiety-like responses in mice evaluated 30 and 60 min after treatment using elevated plus maze (EPM) test. Percentage (%) of open arm entries (**A**,**C**) and % of time spent in the open arms (**B**,**D**) recorded 30 (**A**,**B**) and 60 (**C**,**D**) minutes after 100 mg/kg D2AAK4 injection ((n = 8) and vehicle (n = 8, indicated as 0); i.p). Data are presented as mean ± SEM. The results from the t-test analyses indicate the anxiogenic activity elicited by D2AAK4 30 and 60 min after treatment *** *p* < 0.001 for time spent in the open arms (both 30 and 60 min) and for open arm entries (30 min: * *p* = 0.0272 and 60 min: ** *p* = 0.0092).

To evaluate the influence of the acute administration of D2AAK4 on anxiety-like responses in mice, EPM tests were conducted. The statistical analysis (t-test) of the results for the activity of D2AAK4 (100 mg/kg) on anxiety-like responses indicated significant influence on % of the time spent in the open arms (*p* < 0.0001) (Figure 10) evaluated 30 and 60 min after D2AAK4 administration and % of the open arm entries (*p* = 0.0272 and *p* = 0.0092 after 30 and 60 min, respectively) (Figure 10). In particular, the decrease in both % of the time spent in the open arms and the open arm entries indicates anxiogenic activity of D2AAK4 (Figure 10).

## 4. Discussion

The aim of the presented study was a detailed in vitro, in silico and in vivo characterization of the virtual hit D2AAK4 identified in structure-based virtual screening [9]. Such a characterization of the hit compound is useful prior to its optimization to find a lead molecule for a potential antipsychotic.

D2AAK4 is a multitarget compound with moderate affinity to dopamine D_1_, D_2_, D_3_ and serotonin 5-HT_2A_ and 5-HT_7_ receptors. In particular, D2AAK4 is a low-affinity D_2_ receptor antagonist with moderate (3.67 times higher) affinity for 5-HT_2A_ receptors [9]; that is, with a 5-HT_2A_/D_2_ affinity ratio (*K*_i_ D_2_/*K*_i_ 5-HT_2A_) higher than 1.12 (Meltzer’s ratio) [31], making it a compound with an atypical antipsychotic profile. Atypical antipsychotics such as ziprasidone and paliperidone (9-OH-risperidone, the primary metabolite of risperidone approved by US Food and Drug Administration (FDA) in 2006) show 5-HT_2A_/D_2_ affinity ratios of ~5.60 and 2.16, respectively. Moreover, the affinity of D2AAK4 to serotonin 5-HT_7_ receptor could be beneficial to treat cognitive symptoms of schizophrenia [35]. It also has low affinity to other GPCRs that have been pointed out as off-targets within the antipsychotic pharmacology profile, i.e., histamine H_1_ receptor and muscarinic M_1_ receptor. Histamine H_1_ receptors antagonism is responsible for weight gain associated with second-generation antipsychotics [36] as well as with sedation [37]. M_1_ muscarinic receptor blockade causes memory impairment [38] while activation or positive allosteric modulation of this receptor may be a way to treat cognitive disturbances in schizophrenia [39].

As a multitarget compound, D2AAK4 could be of interest for further optimization of its pharmacological profile in the search of a potential lead compound with atypical antipsychotic properties. The multitarget approach to drug discovery is currently a hot topic in medicinal chemistry. It turned out that in the case of diseases with complex pathomechanisms, such as neurodegenerative diseases, cancer or schizophrenia, a single-target approach is not promising. Modulation of several receptors with one drug molecule also has pharmacokinetic advantages and allows one to avoid drug–drug interactions and to minimize side effects. For instance, the promising multitarget antipsychotic brexpiprazole exerts partial agonism at dopamine D_2_, D_3_ and serotonin 5-HT_1A_ receptors combined with antagonism at 5-HT_2A_, 5-HT_2B_ and 5-HT_7_ receptors [40]. This drug was approved by the FDA in 2015 for schizophrenia and as adjunctive treatment in major depression, and by the European Medicines Agency (EMA) in 2018 for schizophrenia treatment. Side effects of brexpiprazole include weight gain, akathisia, upper respiratory tract infections, somnolence, headache, and nasopharyngitis (as reviewed in [8]).

Importantly, D2AAK4 is an antagonist of both dopamine D_2_ receptor and serotonin 5-HT_2A_ receptor, which is beneficial for its antipsychotic profile. All antipsychotics currently present on the market are dopamine D_2_ receptor antagonists or partial/biased agonists. Thus, it seems that modulation of this receptor is necessary to treat positive symptoms of the disease. Blockade of dopamine D_2_ receptor relates to a number of side effects, including motor disorders from the extrapyramidal system [41] and endocrine system dysregulation [42]. In the case of D2AAK4 with atypical profile, the risk of extrapyramidal disorders would be reduced [43], while a weaker blockade of dopaminergic receptors combined with stronger 5-HT_2A_ blockade is thought to explain the more limited elevation of prolactin plasma levels by second-generation antipsychotics as a group over first-generation antipsychotics [44]. Furthermore, antagonism of 5-HT_2A_ receptor is considered advantageous for the treatment of negative symptoms of schizophrenia [45].

A part of the studies presented here is molecular modeling of interactions of D2AAK4 with respective aminergic GPCRs. In the case of serotonin 5-HT_7_ receptor, first, we constructed a homology model of this protein. Our model is an addition to previously published homology models of serotonin 5-HT_7_ receptor [46,47,48,49] and is in general accordance with them. Molecular interactions of D2AAK4 with the studied aminergic GPCRs are typical for ligands with a protonatable nitrogen atom interacting with the conserved Asp 3.32 as the main anchoring point [50]. Other identified residues, in particular Phe 6.51, Phe 6.52, His 6.55 and the residues from the extracellular loop are in agreement with our earlier results for D2AAK1 [12] and its derivatives [13,23]. The identified interactions include residues (Ser 5.43, Ser 5.46, His 6.55) that have been previously identified as determinants for the specific multitarget binding profile of atypical antipsychotics such as clozapine and olanzapine [11,51]. Moreover, targeted chemical modulation of ligand interaction with residue His 6.55 at D_2_ receptors led to the development of a signaling-pathway-specific antagonist/partial agonism ligand [52]. Overall, the multitarget interactions here described for D2AAK4 provide a robust structural support for rational further developments on this virtual hit.

The compound D2AAK4 turned out to be active in behavioral studies at a dose of 100 mg/kg. It decreased amphetamine-induced hyperactivity in mice, which is a measure of its antipsychotic potential [53]. Moreover, it improved memory consolidation in mice in the passive avoidance test. The PA is based on the acquisition, storage and retention of aversive Pavlovian conditioning involving short- and long-term memory processes. There is evidence showing that the antipsychotics generally showed impairment of the PA response in rodents [54]. In particular, clozapine, olanzapine, lurasidone and asenapine presented impaired PA performance, so it would be desirable to find drug candidates that did not impair memory performance in early screening tests. In addition, risperidone impaired PA performance, but only at doses that were 10-fold greater than those active in the test of inhibition of hyperlocomotion induced by MK-801 predictive of antipsychotic-like activity, while aripiprazole did not impair PA performance but was poorly active in the MK-801 test [54]. On the other hand, multiple studies have shown that the second-generation antipsychotics (SGAs) improved set-shifting ability (cognitive flexibility) (as reviewed in [55]). For instance, clozapine and olanzapine presented positive effects in attention and verbal fluency with improvements in executive functioning and delayed recall, while olanzapine also showed improvement in selective attention, vigilance, verbal learning and memory (as reviewed in [55]).

Current antipsychotics are efficacious at treating positive symptoms, and it is considered that atypical antipsychotics should be generally preferred over first-generation drugs in the treatment of schizophrenia with prominent negative symptoms, and they also appear to be associated with lower cognitive impairment and better functional outcomes [56,57,58]. In particular, first-generation drugs often even worsen the cognitive abilities of the patients, which is one of the reasons for lack of patient compliance with drug regimes. However, the efficacy of atypical antipsychotics against cognitive impairment and negative symptoms is at best limited, according to the most recent effectiveness reviews and pharmacotherapy guidelines [59]. For example, results from the Clinical Antipsychotic Trials of Intervention Effectiveness (CATIE) study (as reviewed in [55]), which included chronic schizophrenia patients, indicated that the improvement in cognition was found to be small [60], with questionable clinical significance [61]. Thus, it is urgently needed to elaborate an antipsychotic able to cure cognitive and negative symptoms as well, as these are still considered important unmet clinical needs. In this context, cariprazine showed efficacy in schizophrenia patients with predominant negative symptoms [62], having been proposed as a good therapeutic option for this population [63].

The EPM test indicated that D2AAK4 has anxiogenic properties in this mice model, which contrasts with the previously published compound D2AAK1 [12]. It was shown that atypical antipsychotics including aripiprazole, quetiapine, olanzapine, and risperidone are helpful in addressing a range of anxiety and depressive symptoms in individuals with schizophrenia [64]. In this regard, optimization of D2AAK4 will have aims—among others—of obtaining compound with anxiolytic characteristics. Prior to this task, the molecular target responsible for the anxiogenic effect needs to be identified, and then molecular modeling approaches may be used to minimize the interactions of D2AAK4 derivatives with this receptor.

## 5. Conclusions

In summary, D2AAK4 is a promising virtual hit, confirmed in vitro and active in vivo, which is a starting point to generate a lead molecule for a novel antipsychotic. Attempts will be made to further balance its multitarget profile as well as to obtain analogs with anxiolytic, not anxiogenic, properties.

## Figures and Tables

**Table 1:** Experimental radioligand binding data for D2AAK4. Data are expressed as p*K_i_* (mean ± SEM) of two (or three for 5-HT_2A_) independent experiments performed in duplicate. ^a^ Full displacement of specific binding was not achieved at the maximum concentration assayed, so p*K*_i_ value could be not accurately estimated; maximum displacement achieved (at 100 μM) was 88% (5-HT_7_) and 91% (M_1_). Clozapine reference affinity values at the indicated targets are shown for comparison. ^b^ Data taken from PDSP Ki database (https://pdsp.unc.edu/databases/kidb.php). ^c^ Data taken from [32].

	p*K*_i_							
Comp.	*h*D_2_	*h*D_1_	*h*D_3_	*h*5-HT_1A_	*h*5-HT_2A_	*h*5-HT_7_	*h*H_1_	*h*M_1_
**D2AAK4**	5.73 ± 0.09	5.60 ± 0.08	5.03 ± 0.08	-	6.31 ± 0.15	5.18 ± 0.24 ^a^	5.41 ± 0.05	5.41 ± 0.11 ^a^
**Clozapine**	6.81 ^b^	6.72 ^b^	6.38 ^b^	6.91 ^b^	8.10 ^b^	6.83 ^c^	8.93 ^b^	8.03 ^b^

## Supplementary Materials

The following are available online at https://www.mdpi.com/2218-273X/10/2/349/s1, Figure S1. Competition binding curves of D2AAK4 in radioligand binding assays at the indicated receptors. Figure S2. Ligand RMSD values in 200 ns molecular dynamics simulations.

## Abbreviations

5-CT	5-carboxamidotryptamine
5-HT	Serotonin
cAMP	Cyclic adenosine monophosphate
CATIE	Clinical Antipsychotic Trials of Intervention Effectiveness
EPM	Elevated plus maze
GABA	γ-aminobutyric acid
GPCRs	G protein-coupled receptors
IP	Inositol phosphate
MD	Molecular dynamics
PA	Passive avoidance
POPC	1-palmitoyl-2-oleoyl-sn-glycero-3-phosphocholine
YLDs	Years lived with disability
