# Supplementary material for: N-(2-Hydroxyphenyl)-1-[3-(2-oxo-2,3-dihydro-1H- benzimidazol-1-yl)propyl]piperidine-4-Carboxamide (D2AAK4), a Multi-Target Ligand of Aminergic GPCRs, as a Potential Antipsychotic
Source: Biomolecules. 2020 Feb 24;10(2):349. doi: 10.3390/biom10020349 (PMC7072648; doi:10.3390/biom10020349)
Supplement: Supplementary file 1 [file biomolecules-10-00349-s001.pdf]

Supplementary Information

N-(2-hydroxyphenyl)-1-[3-(2-oxo-2,3-dihydro-1*H*-benzimidazol-1-yl)propyl]piperidine-4-carboxamide (D2AAK4), a multi-target ligand of aminergic GPCRs, as a potential antipsychotic

Agnieszka A. Kaczor [1,2*], Katarzyna M. Targowska-Duda[3], Andrea G. Silva[4], Magda Kondej[1], Grażyna Biała[5], Marián Castro[4]

[1]Department of Synthesis and Chemical Technology of Pharmaceutical Substances, Faculty of Pharmacy with Division of Medical Analytics, Medical University of Lublin, 4A Chodźki St., PL-20093 Lublin, Poland ; magda.kondej@onet.pl (M.K.)

[2]School of Pharmacy, University of Eastern Finland, P.O. Box 1627, FI-70211 Kuopio, Finland

[3]Department of Biopharmacy, Faculty of Pharmacy with Division of Medical Analytics, Medical University of Lublin, 4A Chodźki St., PL-20093 Lublin, Poland ; katarzyna.duda@umlub.pl (K.T.D.)

[4]Department of Pharmacology, Universidade de Santiago de Compostela, Center for Research in Molecular Medicine and Chronic Diseases (CIMUS), Avda de Barcelona, E-15782 Santiago de Compostela, Spain ; andrea.garcia.silva@rai.usc.es (A.G.S.), marian.castro@usc.es (M.C.)

[5]Department of Pharmacology and Pharmacodynamics, Faculty of Pharmacy with Division of Medical Analytics, Medical University of Lublin, 4A Chodźki St., PL-20093 Lublin, Poland ; grazyna.biała@umlub.pl (G.B.)

* Correspondence: agnieszka.kaczor@umlub.pl; Tel.: +48-81-448-7273

Received: date; Accepted: date; Published: date

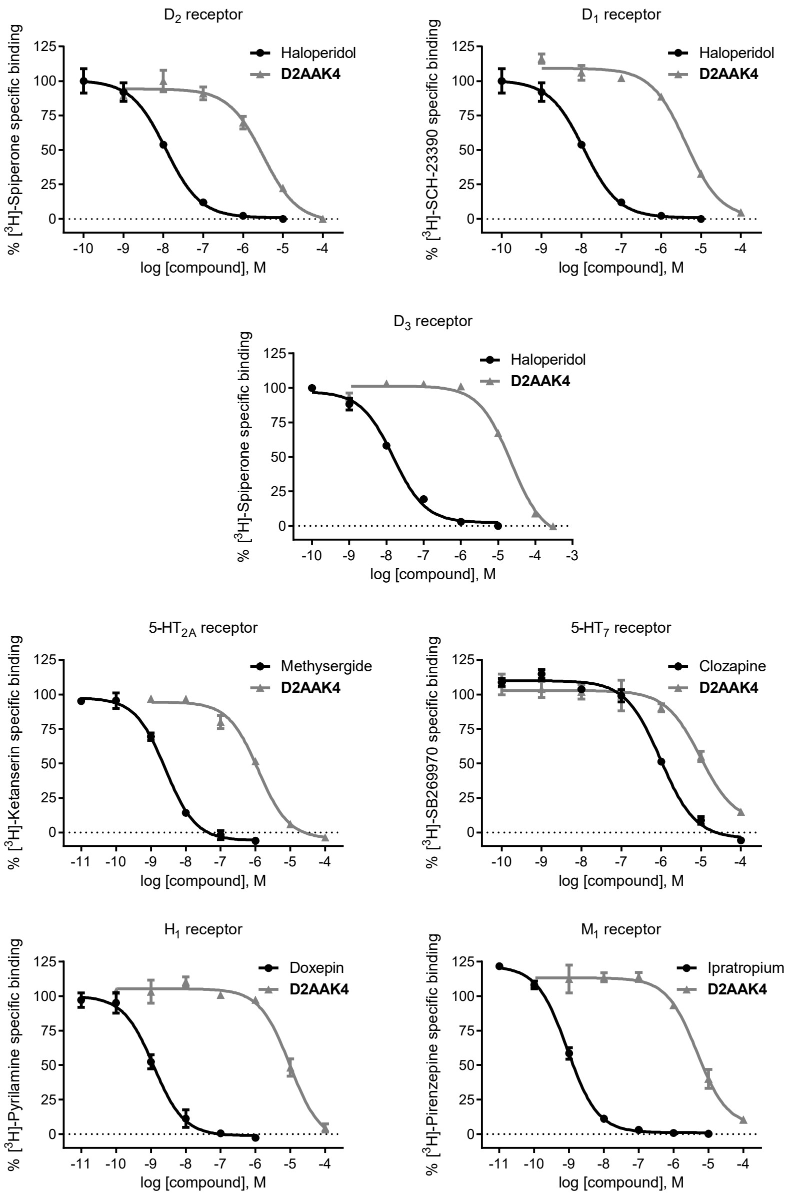

Figure S1. Competition binding curves of D2AAK4 in radioligand binding assays at the indicated receptors. Concentration-dependent displacement of the specific radioligand binding by compound D2AAK4 and the corresponding reference compound at the human cloned receptors indicated. The graphs show the data (mean ± SEM) of a representative experiment out of two (or three for 5-HT_{2A}) independent experiments performed in duplicate. pK_i (mean ± SEM; n = 3 experiments performed in duplicate) and average K_i (nM) values for reference compounds included as internal controls in the binding assays were: haloperidol (D_1) = 8.30 ± 0.07 (5.22 nM); haloperidol (D_1) = 7.93 ± 0.02 (11.8 nM); haloperidol (D_3) = 8.00 ± 0.08 (10.5 nM); 5-carboxamidotryptamine (5-CT) (5-HT_{1A}) = 9.00± 0.06 (1.04 nM); methysergide (5-HT_{2A}) = 9.29 ± 0.07 (0.53 nM); clozapine (5-HT_7) = 6.20 ± 0.05 (632 nM); doxepin (H_1) = 9.26 ± 0.18 (0.55 nM); ipratropium (M_1) = 9.27 ± 0.03 (0.53 nM).

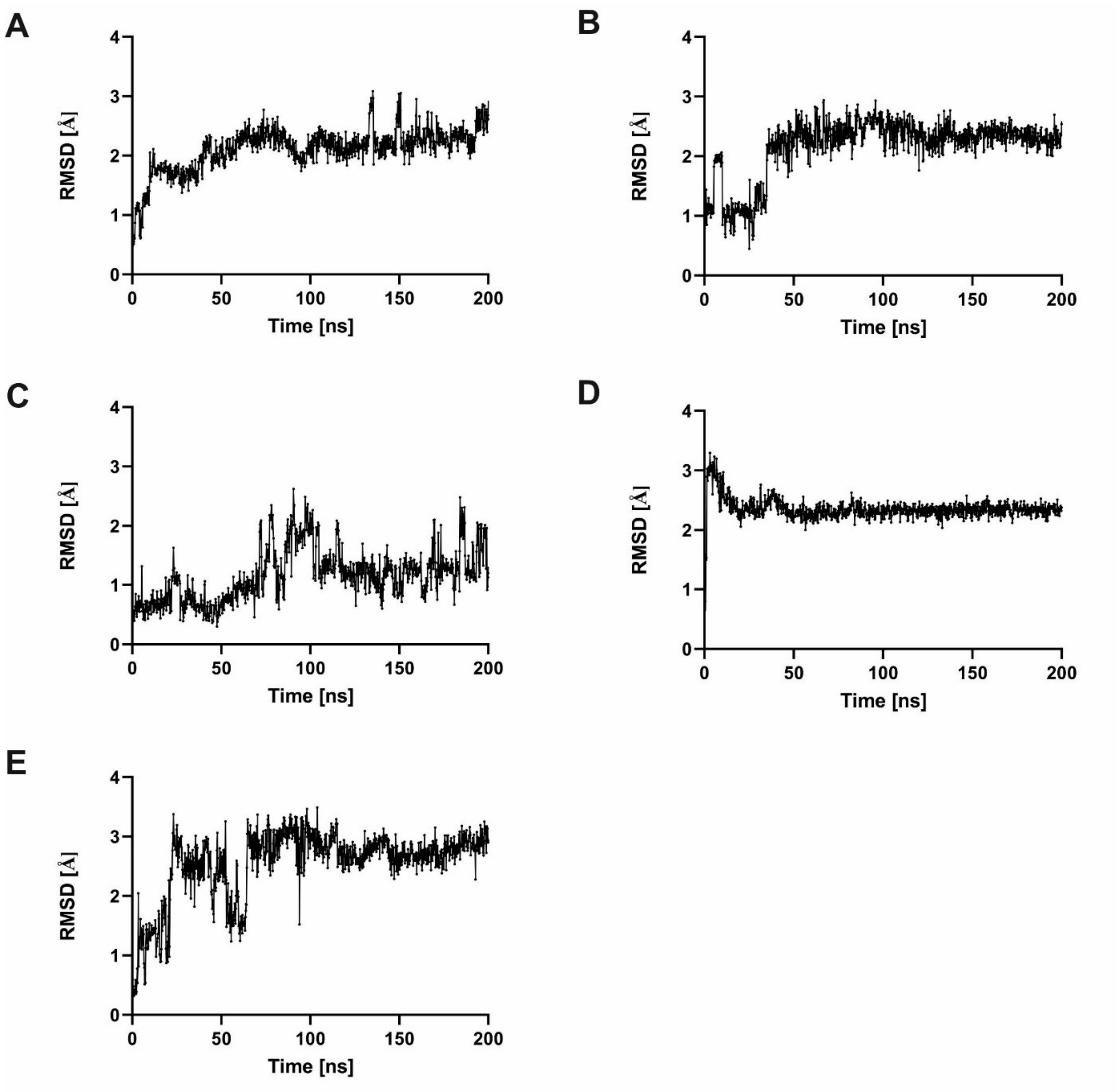

Figure S2. Ligand RMSD values during 200 ns molecular dynamics simulations for D2AAK4 in complex with dopamine D_1 (A), D_2 (B), D_3 (C) and serotonin 5-HT_{2A} (D) and 5-HT_7 receptors (E).

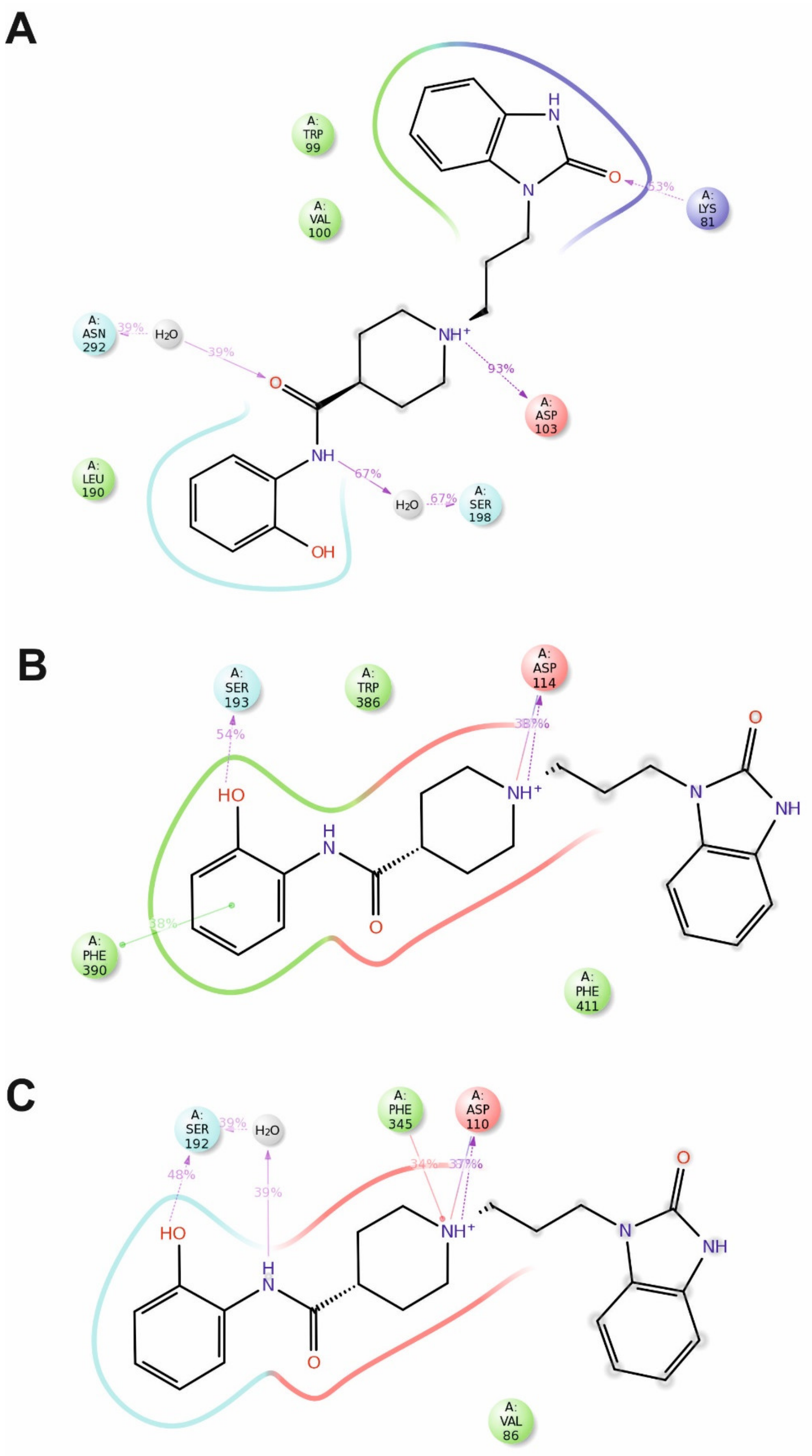

Figure S3. Molecular interactions of D2AAK4 with human dopamine D_1 (A), D_2 (B) and D_3 (C) receptor during 200 ns molecular dynamics simulations: summary of contacts. Interactions that occur more than 30% of the simulation time in the selected trajectory (0 through 200 ns) are shown.

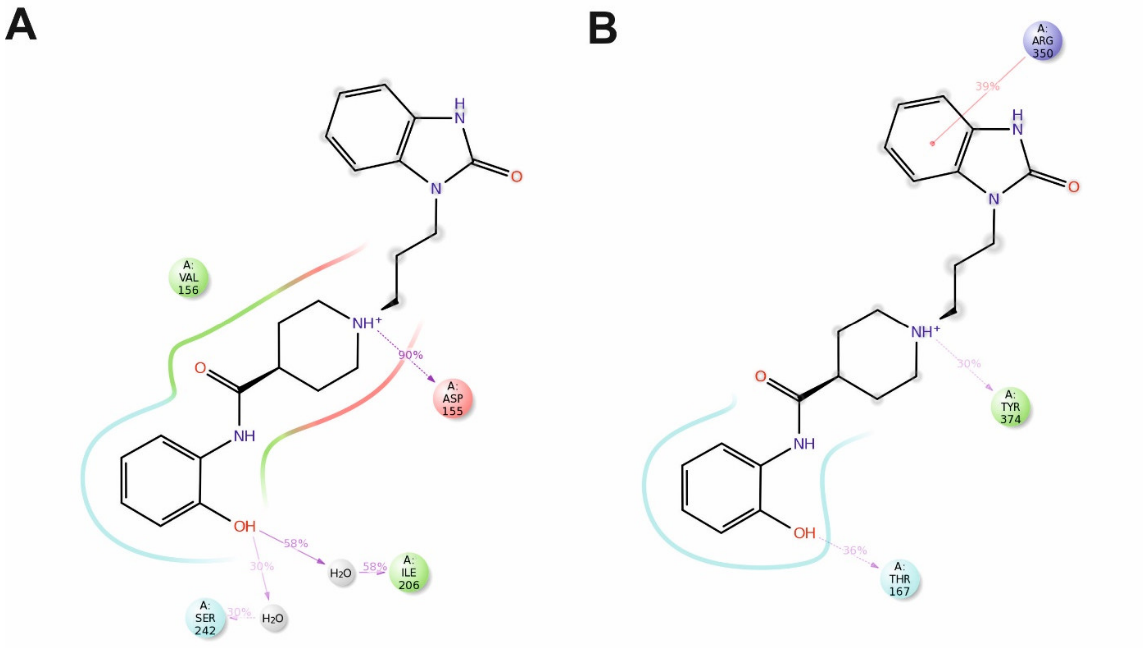

Figure S4. Molecular interactions of D2AAK4 with human serotonin 5-HT_{2A} (A) and 5-HT_7 (B) receptor during 200 ns molecular dynamics simulations: summary of contacts. Interactions that occur more than 30% of the simulation time in the selected trajectory (0 through 200 ns) are shown.

Table S1. Results summary of the evaluation of compound D2AAK4 in *in vitro* functional assays at human cloned D_2 and 5-HT_{2A} receptors. [a] efficacy (% inh., % of inhibition of dopamine response) as D_2 antagonist in cAMP assays; [b] efficacy (% inh., % of inhibition of 5-HT response) and potency (pIC_{50}, -log IC_{50}; IC_{50}, concentration of the compound eliciting the 50% of maximal compound response) as 5-HT_{2A} antagonist in IP assays. Data are mean ± SEM of 2-3 independent experiments performed in duplicate or triplicate.

***h*D2[a] [8]**		***h*5-HT2A[b]**		
**% inh. at 10 μM**	**% inh. at 100 μM**	**% inh. at 10 μM**	**pIC_{50}**	**IC_{50} [nM]**
12.1 ± 6.0%	44.1 ± 1.5%	97.4 ± 1.6%	6.15 ± 0.14	714
